# One-piece extended depth of focus intraocular lens implantation in the capsular bag in the presence of a posterior capsule rupture

**DOI:** 10.1097/RC9.0000000000000376

**Published:** 2026-04-01

**Authors:** Adam Hatoum, Disha Singhania, Kirupakaran Arun, Victoria Cosgrove, Nizar Din, Mukhtar Bizrah

**Affiliations:** a Western Eye Hospital, Imperial College Healthcare Trust, London, UK; b Harley Vision, St John & St Elizabeth Hospital, London, UK

**Keywords:** case report, cataract surgery, extended depth of focus lens, multifocal intraocular lens, posterior capsule rupture, refractive lens exchange

## Abstract

**Introduction and importance:**

Posterior capsule rupture has historically prevented implantation of one-piece intraocular lenses in the bag. We present a case of extended depth of focus (EDOF) intraocular lens insertion into the capsular bag after posterior capsule rupture with an excellent post-operative outcome.

**Case presentation:**

The patient was a 64-year-old female presenting for bilateral refractive exchange to reduce dependence on spectacles. The patient had hypermetropic presbyopia, with no other previous ophthalmic history. She had a non-significant cataract in both eyes. Pre-operative unaided logMAR vision was 0.78 (0.00 pinhole) in her right eye and 0.90 (0.00 pinhole) in her left eye. During right eye surgery, significant posterior pressure was noted throughout, with a shallow anterior chamber. A posterior capsule rupture was noted following phacoemulsification. A one-piece intraocular increased range of focus lens was inserted in the bag with patient consent. It was well centered and stable. On day 13 post-operatively, unaided logMAR vision was 0.0 and N8, and intraocular pressure (IOP) was 10 mmHg. The patient was happy with her visual outcome and the post-operative course was uneventful with week 6 unaided vision being −0.2 and N6 and IOP 10 mmHg and month 6 logMAR 0.0 and IOP 15 mmHg. Visual outcome was similar to the left eye, which did not have a posterior capsular rupture.

**Clinical discussion:**

EDOF lenses allow enhanced intermediate vision while maintaining excellent distance vision with minimal compromise. The advanced optical design may mean they are sensitive to tilt and decentration, but studies show they are less affected than trifocal lenses.

**Conclusions:**

Dependent on careful assessment of the individual case, one-piece intraocular lens implantation into the capsular bag after posterior capsule rupture may be possible. This can be especially useful for premium intraocular lenses, which are often designed as one-piece lenses not suitable for sulcus insertion.

## Introduction

Extended depth of focus (EDOF) and trifocal intraocular lenses (IOLs) have emerged as a transformative option in refractive lens exchange. EDOF lenses have been shown to provide excellent distance, improved intermediate vision, and increased spectacle independence for near tasks in comparison to monofocal lenses. Though near acuity is worse than trifocal IOLs, dysphotopsia and contrast sensitivity are typically better dependent on the design^[^1^]^.

HIGHLIGHTS

- Posterior capsule rupture during cataract surgery typically contraindicates one-piece intraocular lens insertion into the capsular bag.
- Extended depth of focus (EDOF) lenses must be well centered and balanced upon implantation to ensure the desired visual outcome.
- We have shown that in careful case selection, EDOF lens can be implanted into the capsular bag despite posterior capsule rupture, leading to fantastic post-operative results.

Posterior capsular rupture (PCR) during cataract surgery can pose significant challenges to their implantation, as good capsular support and centration are vital for multifocal optics^[^2^]^. In routine cataract surgery, implantation of a sulcus lens can provide good distance visual acuity – especially with optic capture^[^3^]^ – but in patients presenting for refractive lens exchange with good distance visual acuity already, continued spectacle dependence for intermediate and near could lead to significant patient dissatisfaction.

This case report demonstrates the ability to insert an EDOF IOL into the bag after PCR in a patient having refractive lens exchange for hypermetropic presbyopia, demonstrating that a premium one-piece IOL can potentially be safely and effectively implanted into the bag. By reporting this case, we hope to increase awareness of this possibility and its success in a carefully selected situation.

This work has been reported in line with the SCARE criteria^[^4^]^.

## Case presentation

The patient was a 64-year-old female presenting with hypermetropia and mild astigmatism resulting in poor unaided distance and near vision. She wanted to reduce her dependence on visual aids (full-time varifocal spectacle use with occasional contact lens use).

She had no relevant ophthalmic, medical, or family history and no previous interventions regarding her symptoms. She is employed, a current driver, and enjoys walking and antique dealing.

Her visual acuity was 0.78 unaided (0.00 best corrected and pinhole) in her right eye and 0.90 unaided (0.00 best corrected and pinhole) in her left eye. Near vision was N4 with an add of +2.50 in both eyes (unaided near vision is not available). Intraocular pressure (IOP) was 16 mmHg in the right eye and 17 mmHg in the left eye. Her manifest refraction was +3.50/−0.50 × 60 add +2.50 in her right eye and +4.00/−0.50 × 100 add +2.50 in her left eye. She had a non-significant cataract in both eyes, a small right eye choroidal nevus, and an otherwise normal anterior and posterior segment examination (including healthy optical coherence tomography imaging of the macula). Axial length was 23.61 (right) and 23.72 (left).

The recommended procedure was bilateral refractive lens exchange. Following a discussion of benefits and risks, the patient consented to the procedure and opted for an increased range of focus IOL (J&J TECNIS PureSee™ IOL).

**Figure 1.**
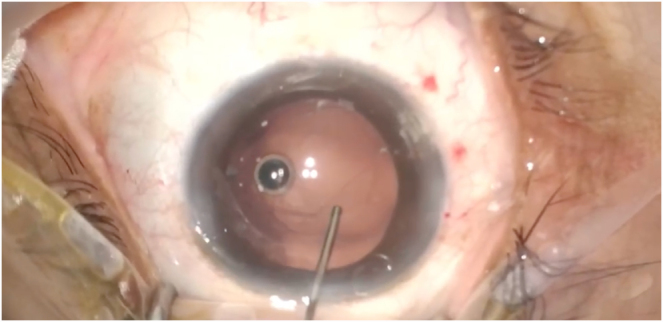
Intraoperative microscopic view of posterior capsule rupture prior to lens insertion.

Throughout phacoemulsification, the right eye had significant posterior pressure with a shallow anterior chamber and posterior capsular elevation. A posterior capsule rupture was noted extending horizontally from 3 o’clock to 9 o’clock, margins visualized (Fig. 1). The patient was informed and given the options of monofocal lens insertion into the sulcus (standard) or EDOF one-piece lens insertion into the bag, noting risk of further surgery with the latter. The patient chose a trial of EDOF lens insertion into the bag.

Triamcinolone-assisted anterior vitrectomy was performed due to recognition of vitreous loss, and the planned J&J TECNIS PureSee™ IOL was inserted into the bag. It was well centered and stable. Miochol was injected and post-operative drops of tobramycin and dexamethasone (tobradex) four times a day and nepafenac (nevanac) three times a day were given for 4 weeks.

The left eye was operated on thereafter (same day) and had identical significant posterior pressure as soon as phacoemulsification started. The surgeon therefore decided it was safer to remove the soft lens using bimanual irrigation and aspiration (no phacoemulsification). This was successful in avoiding a PCR, and the same EDOF (J&J TECNIS PureSee™ IOL) IOL was inserted into the bag with no complications.

The patient was assessed on day 1 post-operatively. The unaided vision was 0.0 and N8 in the right eye. There was minimal corneal edema as expected on day 1 and AC cells 0.5+, with IOL stable in the bag. IOP was 32 mmHg and dorzolamide and timolol twice a day was prescribed. The unaided vision was 0.0 and N8 and IOP was 10 mmHg on day 13 post-operatively (off any IOP-lowering medication for a few days).

**Figure 2.**
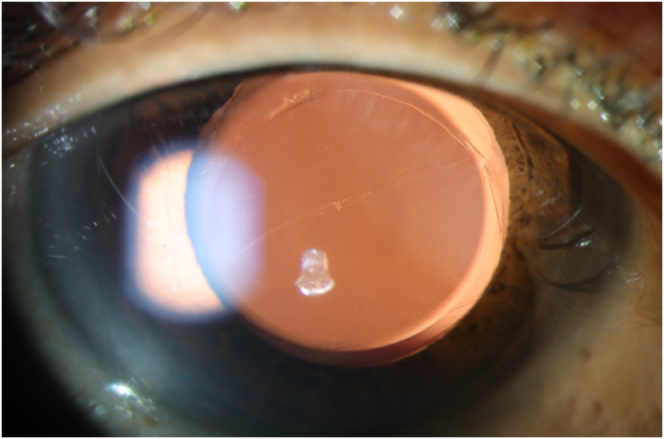
Clinical photograph at week 6 post-operatively demonstrating well-centered IOL in the bag.

At week 6, the IOL was well centered and stable (Fig. 2). The right eye UDVA (uncorrected distance visual acuity) was logMAR 0.2 and the left eye UDVA was logMAR 0.0. UNVA (uncorrected near visual acuity) was N6 (J5) in the right eye and N5 (J3) in the left eye. IOP was 10 mmHg (right) and 11 mmHg (left) at week 6. At month 6, unaided vision was 0.0 in both eyes and IOP was 15 and 14 mmHg in the right and left eyes, respectively.

The patient was happy with her visual outcome and had functional spectacle independence. The attached video shows the walk-through of the case with voice-over from the operating surgeon (Supplemental Digital Content Video 1, available at: http://links.lww.com/IJSCR/A29).

## Discussion

Increased and full range of vision IOLs represent a significant advancement in presbyopia-correcting technology, designed to improve post-operative visual performance across a range of distances^[^5,6^]^.

First approved by the FDA in 2016, EDOF lenses use specialized optical designs to increase the depth of focus, enhancing intermediate vision while maintaining excellent distance acuity with minimal compromise. To standardize the definition and performance expectations for EDOF IOLs, the American Academy of Ophthalmology (AAO) Task Force established consensus criteria, ensuring consistency in clinical evaluation and patient outcomes^[^7^]^. The Concerto Study, a landmark international multicenter case series, demonstrated the efficacy of bilateral EDOF IOL implantation, achieving functional vision at all distances with low rates of dysphotopsia and high patient satisfaction^[^8^]^. This underscores their relevance in modern cataract surgery, especially as daily activities increasingly involve intermediate tasks like using computers and smartphones.

The centration and stability of EDOF IOLs are critical for optimal performance^[^9^]^. Unlike monofocal IOLs, EDOF lenses rely on advanced optical designs such as echelette diffraction gratings or wavefront-shaping technologies to create an extended range of clear vision. These designs are highly sensitive to tilt or decentration, which can degrade contrast sensitivity and induce visual disturbances like glare or halos. Precise alignment with the visual axis is essential for these lenses to function effectively, making in-the-bag fixation the preferred implantation method.

Interestingly, despite the above, some studies suggest that EDOF IOLs are less affected by tilt and decentration compared to trifocal IOLs^[^10^]^. The TECNIS PureSee^TM^ is designed to have a continuous power change rather than low add diffractive in multifocal lenses^[^11^]^. This increased tolerance supports their usability in challenging cases while maintaining high-quality intermediate and distance vision.

PCR is a well-documented complication encountered during cataract surgery. In cases of PCR, surgeons often opt for monofocal IOLs or sulcus placement of three-piece IOLs due to concerns about lens stability^[^12^]^. However, sulcus placement of EDOF lenses is generally contraindicated because they are one-piece IOLs that cause iris chafing, pigment dispersion, and risk of uveitis–glaucoma–hyphema syndrome^[^13^]^.

Given the relative novelty of EDOF lenses, a higher volume of literature is available regarding monofocal IOL placement in the bag in the presence of a PCR. A 2021 paper by Masayuki presented the long-term clinical outcomes of monofocal IOL in-the-bag fixation in eyes with PCR^[^12^]^. They described their eligibility requirements as follows: (1) continuous curvilinear capsulorhexis was intact; (2) lens nuclear fragment, lens cortex, and vitreous were adequately treated; and (3) posterior capsule was partially retained in both directions, allowing us to embed two IOL haptics while injecting an ophthalmic viscosurgical device between the anterior and posterior capsules. This retrospective case series of 11 eyes verified that in-the-bag IOL implantation in eyes with PCR can yield good clinical outcomes when certain criteria are satisfied. Patients showed refractive predictability comparable with normal eyes at 1-year post-op, and a 1D myopic shift at 5 years post-operatively, compared to that at 3 months with sulcus fixation.

In our case report, successful anterior vitrectomy preserved capsular integrity sufficiently to allow secure in-the-bag placement of an EDOF lens. This approach ensured both physical stability and optimal visual outcomes, highlighting that premium lens implantation remains feasible even in complex surgical scenarios when meticulous techniques are employed. A literature search identified a similar case report by Srinivasaraghavan *et al*, which also describes the successful implantation of an EDOF IOL in a patient with a PCR^[^14^]^. This patient, a young individual with a traumatic cataract and isolated PCR, underwent cataract extraction and anterior vitrectomy. As with our case, the surgical team achieved stable in-the-bag placement of an EDOF IOL, with favorable post-operative outcomes. This case again underscores the potential for successful EDOF IOL implantation in selected cases of PCR.

## Conclusion

This case demonstrates the possibility of inserting a one-piece intraocular (EDOF) lens in the bag after posterior capsule rupture. This allows the use of special lenses such as IROF (increased range of focus) lenses in these patients. It is important to carefully assess the individual case for suitability of this, but when appropriate, this option can optimize outcomes and allow for increased patient satisfaction, in particular when patients are keen to reduce dependence on visual aids. Further studies are required to assess the long-term outcomes including refractive status of these patients.

## Data Availability

Not applicable.

## Research registration unique identifying number (UIN)

As this is a case report, it has not been registered as it does not fall under the remit of registration of research studies.
